# Developing High‐Efficiency Electroporation Protocols for Hard‐To‐Transform *Halomonas* spp.

**DOI:** 10.1111/1751-7915.70285

**Published:** 2025-12-21

**Authors:** André A. B. Coimbra, Ian J. White, Leonardo Rios‐Solis

**Affiliations:** ^1^ Department of Biochemical Engineering, Bernard Katz Building University College London London UK; ^2^ Laboratory for Molecular Cell Biology University College London London UK

## Abstract

*Halomonas* species have recently emerged as attractive candidates for next‐generation industrial biotechnology, due to their ability to thrive under high‐salt conditions, which allows for fermentation under open, unsterile conditions. However, their genetic manipulation has long been hindered by difficulties in genetic transformation. In this study, we report the development of a highly efficient electroporation protocol for *Halomonas elongata* DSM 2581. By optimising competent cell preparation and electroporation parameters, and using plasmid DNA purified from *dam*/*dcm* methylation‐deficient *Escherichia coli* , we achieved a maximum transformation efficiency of 2.8 × 10^8^ ± 0.2 × 10^8^ CFU/μg DNA—the highest efficiency reported for any *Halomonas* species to date. Notably, we observed that growing cells in low‐salt medium and harvesting them at late‐stationary phase considerably improved electroporation efficiency. Moreover, we further demonstrated that the use of non‐methylated plasmids helped evade the defence systems of *H. elongata* DSM 2581 that target foreign DNA. Importantly, the protocol proved transferable to other industrially relevant strains, achieving efficiencies of 5.3 × 10^7^ ± 0.1 × 10^7^ and 5.4 × 10^6^ ± 1.2 × 10^6^ CFU/μg DNA in *Halomonas boliviensis* LC1 and *Halomonas campaniensis* LS21, respectively. Altogether, this work establishes a robust, high‐efficiency electroporation method for *Halomonas* spp., facilitating future genetic manipulation and strain engineering work, as well as encouraging further research into underexplored *Halomonas* species.

## Graphical abstract

Genetic manipulation of *Halomonas* species has been limited by poor transformation efficiencies. Here, we establish a highly efficient electroporation protocol for *Halomonas elongata* DSM 2581. This method also proved transferable to other *Halomonas* strains, encouraging further genetic manipulation work in underexplored species.

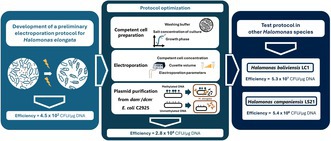

## 1 Introduction

*Halomonas* species have recently emerged as promising chassis for next‐generation industrial biotechnology (Coimbra et al. 2025). *Halomonas* are Gram‐negative bacteria commonly found in high‐saline environments, such as oceans, salt lakes and marshes (Setati 2010). Their ability to thrive under high salt conditions, in which most conventional microorganisms cannot survive, allows them to be cultured under open, unsterile and continuous conditions using wastewater or seawater (Yin et al. 2015; Tan et al. 2011). This constitutes a major advantage over traditional microbial chassis, which require elaborate, energy‐intensive and expensive sterilisation procedures to minimise contamination risks, which also often limit the fermentation type to discontinuous processes, such as batch and fed‐batch fermentations (Trisrivirat et al. 2020; Junker et al. 2006; Zhang et al. 2024). Beyond their favourable chassis properties, many *Halomonas* species, such as *H. boliviensis* LC1, *H. venusta* KT832796 and *H. bluephagenesis* TD01, have demonstrated a remarkable ability to accumulate large amounts of polyhydroxybutyrate (PHB), making them promising candidates for bioplastic production (Tan et al. 2011; Quillaguamán et al. 2008; Stanley et al. 2018). In addition to PHB, multiple *Halomonas* species have been reported to produce high quantities of the osmoprotectants ectoine and hydroxyectoine, high‐value compounds widely used in the cosmetic industry (Coimbra et al. 2025). For instance, *H. elongata* DSM 2581 has been observed to naturally produce 12.91 g/L of ectoine with a productivity of 1.13 g/L/h in a 5‐L bioreactor (Yu et al. 2022).

While these characteristics position *Halomonas* as a promising chassis for next‐generation industrial biotechnology, it is fundamental that reliable and standardised genetic manipulation tools exist, so that chassis robustness and production titers can be improved through cell engineering. However, the poor amenability of *Halomonas* species to being transformed has proved to be one of the main bottlenecks hindering advances in their synthetic biology tool development.

The development of electroporation and chemical transformation protocols in *Halomonas* has been severely limited by their dense cell membrane and extensive surface‐loaded exopolysaccharides (Ye and Chen 2021; Wang et al. 2021; Xu et al. 2022). As such, most studies have resorted to RP4‐mediated conjugation for the transformation of plasmids into *Halomonas* (Xu et al. 2024; Coimbra et al. 2025). However, conjugation is relatively low‐throughput and is restricted to conjugative or mobilisable plasmids (Xu et al. 2022; Ye and Chen 2021). Nevertheless, a few relatively successful attempts have been reported in recent years. In 2016, Harris et al. first reported an electroporation protocol for *Halomonas* sp. O‐1, from which an efficiency of 10^4^ transformants per μg of DNA was obtained. However, such efficiency levels are still significantly lower than those of bacteria such as *E. coli* and *P. aeruginosa* , where transformants are obtained in the order of 10^7^–10^9^ transformants per μg DNA (Calvin and Hanawalt 1988; Choi et al. 2006). In addition, this method showed limited reproducibility in other *Halomonas* strains (Xu et al. 2022; Tsuji et al. 2022). Likewise, an efficiency of only 400 transformants per μg of DNA was reported in an extracellular polysaccharide mutant of *H. bluephagenesis* with improved membrane permeability (Xu et al. 2022). More recently, Park et al. (2024) successfully electroporated *Halomonas* sp. YLGW01 by pre‐treating the cells with CaCl_2_ (50 mM) prior to sucrose (200 mM) washes, but no details were provided regarding the efficiency of the process. Moreover, while Jung et al. (2024) electroporated *Halomonas* sp. YK44 with an efficiency of approximately 10^3^ transformants per μg of DNA, they also reported that they were unable to electroporate *H. shengliensis* , *H. sulfidoxydans* and *H. elongata* , one of the most well‐studied *Halomonas* species (Coimbra et al. 2025).

In this study, we developed a high‐efficiency electroporation protocol for *Halomonas elongata* DSM 2581. This was achieved by optimising electrocompetent cell generation and electroporation parameters, as well as investigating the influence of DNA methylation on transformation efficiency. The optimised protocol was subsequently used to efficiently electroporate the industrially relevant strains *H. boliviensis* LC1 and *H. campaniensis* LS21, thereby demonstrating its broader applicability to other *Halomonas* species.

## 2 Materials and Methods

### 2.1 Strains and Plasmids

*Halomonas elongata* DSM 2581, *Halomonas boliviensis* LC1 and *Halomonas campaniensis* LS21 were obtained from DSMZ. *Escherichia coli* 10‐beta and *E. coli* C2925 (*dam* ^*−*^, *dcm* ^*−*^) were sourced from New England Biolabs (NEB). Plasmids pSEVA241 (pRO1600/ColE1, Kan^R^) and pSEVA231 (pBBR1, Kan^R^) were kindly provided by Prof. Victor de Lorenzo (Consejo Superior de Investigaciones Científicas).

### 2.2 Media

*E. coli* strains were grown in Luria‐Bertani (LB; Sigma‐Aldrich) medium at 37°C, 250 rpm. *Halomonas* strains were grown in LB medium (0.5% NaCl, pH 7.5) supplemented with 0.5% (LB10), 1.5% (LB20) or 5.5% (LB60) NaCl (Thermo Fisher Scientific). *H. elongata* DSM 2581 and *H. boliviensis* LC1 were incubated at 30°C, 225 rpm; and *H. campaniensis* LS21 at 37°C, 225 rpm.

### 2.3 Growth Curve Analysis

100 μL of an overnight culture of *H. elongata* DSM 2581 in LB60 was transferred into a 50 mL Falcon tube containing 5 mL of LB60 and incubated for 24 h at 30°C, 225 rpm. Culture turbidity was determined by optical density measurements at 600 nm (OD_600_) taken every 1.5 h using a Jenway 7315 UV/visible spectrophotometer. Three biological replicates were performed.

### 2.4 Cell Viability Assays

100 μL of an overnight culture of *H. elongata* DSM 2581 in LB60 was transferred into a 50 mL tube containing 5 mL of LB60, and incubated for 8 h at 30°C, 225 rpm. Cells were harvested (6000 × *g*; 10 min), and the pellets treated for 5 min with either CaCl_2_ (50 mM; Sigma‐Aldrich) or CaCl_2_ (50 mM) + NaCl (6%). Cells were then washed three times (5, 3, 3 mL) with sucrose (300 mM; Fisher Scientific). At each step, samples were collected and diluted 10^6^‐fold in saline (6%). 100 μL of the diluted sample was plated onto LB60 agar plates, and incubated for 48 h at 30°C. The survival rate was calculated by comparing the number of colony‐forming units (CFU) from treated samples to the untreated control.

### 2.5 Preparation of *Halomonas* Electrocompetent Cells

Overnight cultures were subcultured (100 μL) into 5 mL LB10, LB20 or LB60, and incubated for 6, 14 or 24 h at 30°C ( *H. elongata* and *H. boliviensis* ) or 37°C ( *H. campaniensis* ), and at 225 rpm. Electrocompetent cells were prepared at either 4°C or room temperature. Cells were harvested (6000 × *g*; 10 min), and the pellets treated for 5 min with either CaCl_2_ (50 mM) or CaCl_2_ (50 mM) + NaCl (1%, 2% or 6%). Cells were then washed twice (5, 3 mL) with sucrose (300 mM) or glycerol (10%; Sigma‐Aldrich). Cells were resuspended in the same buffer to an OD_600_ of 60, 40, 20 or 10.

### 2.6 Electroporation of *Halomonas*

Electroporation was performed in 0.2‐cm gap cuvettes (Bio‐Rad) with either 40 μL or 200 μL of electrocompetent cells mixed with plasmid DNA. pSEVA241 and pSEVA231 were purified from either *E. coli* 10‐beta, *E. coli* C2925 or *Halomonas elongata* DSM 2581 using the E.Z.N.A. Plasmid Mini Kit I (Omega Bio‐Tek), as per the manufacturer's instructions. Electroporation was conducted using either a Bio‐Rad MicroPulser (1.5 kV; 1.7 kV; 2.1 kV; 2.5 kV; 2× 2.1 kV; 2× 2.5 kV; 2× 2.75 kV; 2× 3.0 kV) or a Bio‐Rad Gene Pulser (25 μF; C1 (2.1 kV, 1 pulse, 600 Ω), C2 (2.1 kV, 2 pulses, 800 Ω), C3 (2.3 kV, 3 pulses, 600 Ω), C4 (2.3 kV, 1 pulse, 800 Ω), C5 (2.3 kV, 2 pulses, 600 Ω), C6 (2.3 kV, 3 pulses, 800 Ω), C7 (2.5 kV, 1 pulse, 800 Ω), C8 (2.5 kV, 3 pulses, 600 Ω)). Electroporated cells were then recovered in 1 mL of LB20 or LB60 supplemented with glucose (20 mM; Millipore) for 90 min at 30°C ( *H. elongata* and *H. boliviensis* ) or 37°C ( *H. campaniensis* ), at 225 rpm. Following recovery, cells were plated onto LB20 or LB60 agar supplemented with 15 μg/mL or 150 μg/mL of kanamycin (Thermo Fisher Scientific), respectively, at the appropriate temperature, to allow for colony formation. Electroporation efficiency was calculated according to the following formula:

$$\begin{array}{c} \text{Electroporation efficiency}\;\left(\mathrm{CFU}/\mathrm{\mu g}\;\mathrm{DNA}\right)=\\ \mathrm{CFU}\left(\#\right)\times D_{f}\times \frac{\text{Total volume}\;\left(\mathrm{\mu L}\right)}{\text{Plated volume}\;\left(\mathrm{\mu L}\right)}\times \frac{1000}{\mathrm{DNA}\;\text{amount}\;\left(\mathrm{ng}\right)} \end{array}$$

where *CFU* is the colony count and *D*_*f*_ is the dilution factor.

### 2.7 Transmission Electron Microscopy

Overnight cultures of *H. elongata* DSM 2581 were subcultured (100 μL) into 5 mL of LB60, and incubated for 14 or 24 h at 30°C, 225 rpm. Samples were centrifuged (6000 × *g*; 3 min) to pellet the bacteria, which were then resuspended in a pre‐warmed saline solution of a concentration corresponding to their original growth media. Bacteria were then fixed by the addition of 1/4 final volume of 4× strength fixative solution containing 8% formaldehyde/6% glutaraldehyde (TAAB Laboratories Equipment Ltd) in 0.4 M sodium cacodylate buffer for 2 h at room temperature (RT). Cells were again pelleted and resuspended in fixation solution containing 2% formaldehyde/1.5% glutaraldehyde in 0.1 M sodium cacodylate, and incubated overnight at 4°C.

Samples were then prepared for Transmission Electron Microscopy (TEM) using a modified protocol (Deerinck et al. 2010). Briefly, samples were washed in 0.1 M sodium cacodylate buffer and post‐fixed in 1% osmium tetroxide/1.5% potassium ferricyanide for 60 min at 4°C. Samples were then washed in distilled H_2_O and treated with 1% thiocarbohydrazide for 20 min at RT, 2% osmium tetroxide for 30 min at RT, 1% uranyl acetate overnight at 4°C, and lead aspartate for 30 min at 60°C, with intermediate washing in distilled H_2_O and re‐pelleting by centrifugation between each step. This was followed by sample dehydration via increasing concentrations of ethanol: 70%, 90% and 100% (x2). Samples were then infiltrated twice with propylene oxide (PO) for 10 min, then with a 2:1 mix of PO:epon resin for 90 min, a 1:1 PO:epon resin for 90 min, 100% epon resin for 2 h, 100% epon resin overnight, and fresh 100% resin for 5 h the following day. Samples were transferred one final time into fresh epon resin, centrifuged to ensure a pellet, and incubated at 60°C overnight to allow for polymerisation.

Embedded samples were polished to a flat surface using a glass knife on an ultramicrotome (UC7 Leica), before a block face was trimmed, and ultrathin 70 nm sections cut using a diamond knife (DiATOME). Sections were collected on 1 × 2 mm Formvar‐coated copper slot grids for TEM imaging. Images were acquired using a Hitachi HT7800 TEM using a Qedira CCD camera (EMSIS). Cell wall thickness and cell diameter measurements were performed using RADIUS software (EMSIS).

### 2.8 Statistical Analysis

Data is presented as mean ± standard deviation, unless stated otherwise. Statistical significance between two groups was assessed using two‐tailed unpaired *t*‐tests. For multiple group comparisons, a one‐way analysis of variance (ANOVA) was performed to evaluate differences amongst group means. For the experiment comparing electroporation efficiencies of *H. elongata* DSM 2581 at different salt concentrations, a lognormal one‐way ANOVA was used to account for the severe heteroscedasticity observed in the residuals. Post hoc Tukey's Honestly Significant Difference tests were conducted to identify specific group‐to‐group differences. For the electroporation parameter optimisation experiments in *H. elongata* DSM 2581, a post hoc Dunnett's multiple comparisons test was used to determine significant differences relative to the 2× 2.5 kV (MicroPulser) condition used in the default protocol. Data and statistical analysis were performed using GraphPad Prism 10.2.3.

## 3 Results

### 3.1 Establishing a Baseline Electroporation Protocol

To establish a functional electroporation protocol for *H. elongata* DSM 2581, we first attempted the transformation of plasmid pSEVA241, a vector available from the Standard European Vector Architecture (SEVA) (Durante‐Rodríguez et al. 2014). This plasmid harbours the broad‐host range, high‐copy number origin of replication pRO1600/ColE1, previously shown to be stable in other *Halomonas* species (Shu et al. 2023; Liu et al. 2023). In addition, it carries a kanamycin resistance gene, which has previously been successfully used as a selection marker in *H. elongata* (Tanimura et al. 2013; Rodríguez‐Sáiz et al. 2007).

**FIGURE 1:**
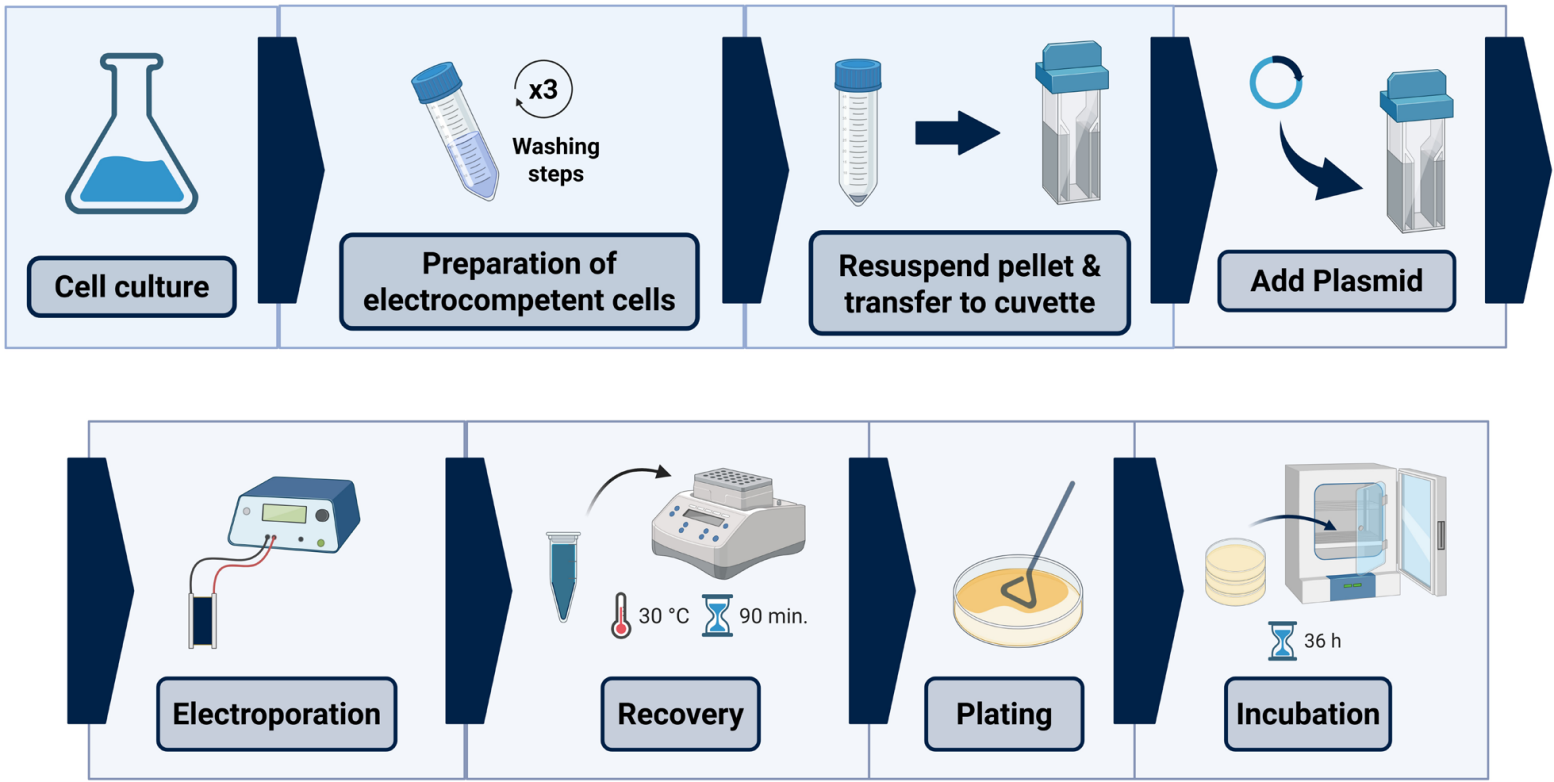
Preliminary electroporation protocol of *Halomonas elongata* DSM 2581. Cells were cultured in LB medium supplemented with NaCl (LB60) at 30°C and 225 rpm for 14 h. Electrocompetent cells were prepared by washing the harvested cells with CaCl_2_ (50 mM) + NaCl (6%) for 5 min, followed by two sucrose (300 mM) washes. The cell pellet was then resuspended in sucrose and transferred to a 0.2‐cm gap electroporation cuvette, to which the plasmid DNA was subsequently added. Electroporation was performed using a Bio‐Rad MicroPulser, after which cells were recovered in LB60 supplemented with glucose (20 mM) at 30°C and 225 rpm for 90 min. Following recovery, electroporated cells were plated onto LB60 agar plates and incubated at 30°C for 36 h to allow for colony formation. (Created in BioRender. Rios Solis, L. (2025) https://BioRender.com/osb8xfd).

The primary objective at this stage was to establish a baseline electroporation protocol that would serve as a starting point for further optimisation. Accordingly, we based our preliminary method for generating electrocompetent cells on the protocols established by Harris et al. (2016) and Park et al. (2024), for *H*. sp. O‐1 and *H*. sp. YLGW01, respectively (Figure 1). This entailed first growing the cells to early‐stationary phase (Figure S1), at which point cells were harvested and treated with CaCl_2_ (50 mM). To mitigate osmotic stress, which we observed to significantly reduce cell viability (Figure S2), the CaCl_2_ solution was supplemented with NaCl (60 g/L). Cells were then washed twice with sucrose (300 mM) before being resuspended in the same buffer and transferred to an electroporation cuvette, to which pSEVA241 was subsequently added.

### 3.2 Preliminary Electroporation Tests

**FIGURE 2:**
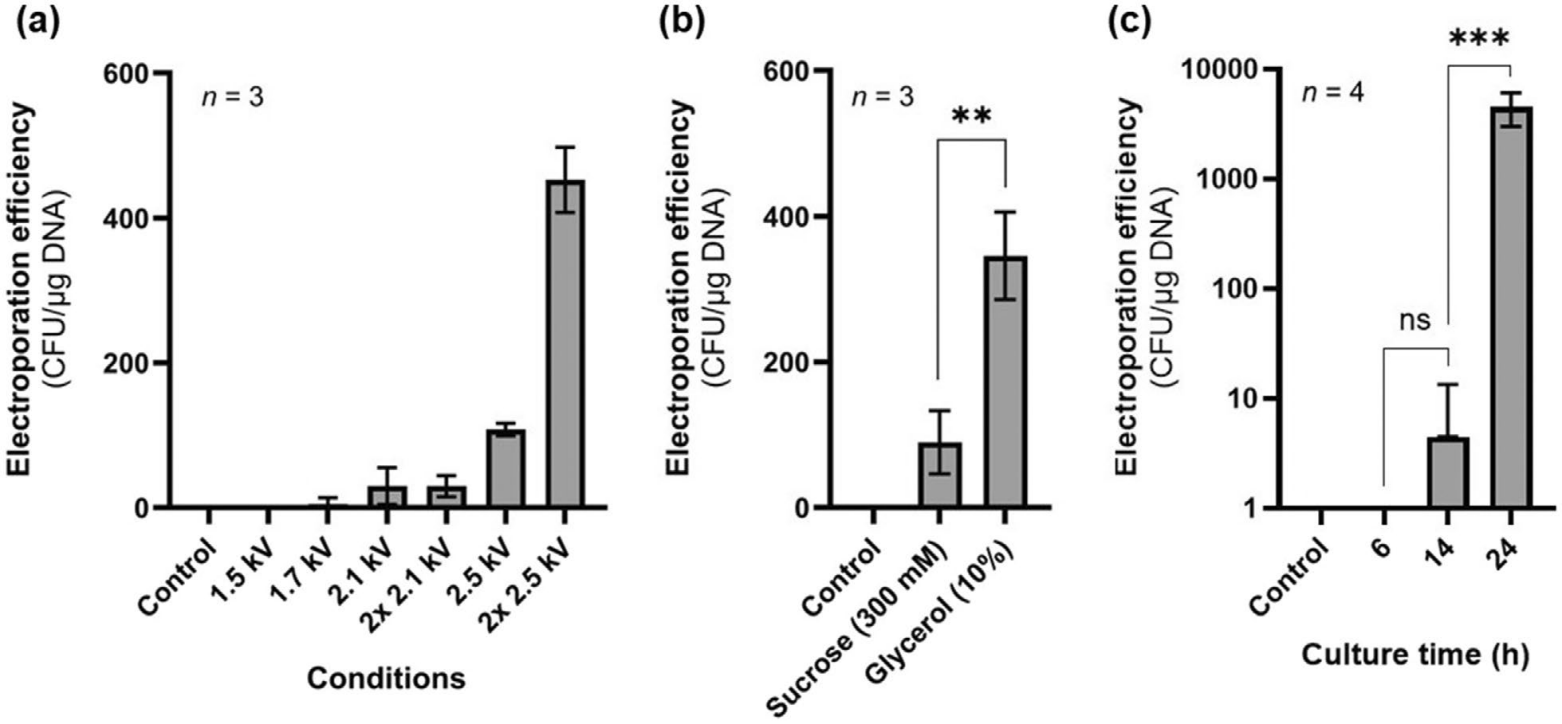
Preliminary electroporation tests of *Halomonas elongata* DSM 2581. (a) Electroporation efficiencies at different voltages (1.5, 1.7, 2.1 and 2.5 kV) and pulse numbers (2× 2.1, 2× 2.5 kV). (b) Electroporation efficiencies from electrocompetent cells prepared with sucrose (300 mM) vs. glycerol (10%). (c) Electroporation efficiencies from electrocompetent cells prepared from cells harvested at 6, 14 and 24 h. Data shown represent mean ± standard deviation from three biological replicates for panels (a) and (b), and four biological replicates for panel (c). Negative control experiments were performed by electroporating electrocompetent cells without the addition of plasmid pSEVA241. (ns, not significant; ***p < 0.001)

To evaluate whether the preliminary protocol was capable of successfully electroporating pSEVA241 into *H. elongata* , we first pulsed the electrocompetent cells across a range of different voltages and pulse numbers (Figure 2a). While the protocol proved functional, the observed electroporation efficiencies were modest. Notably, efficiencies seemed to be improved with both increasing voltage and pulse number, with the highest efficiency recorded at 4.5 × 10^2^ ± 0.4 × 10^2^ CFU/μg DNA, when cells were pulsed twice at 2.5 kV. Moreover, these results not only validated our preliminary electroporation method, but also indicated that the plasmid origin of replication pRO1600/ColE1 can be stably maintained in *H. elongata* DSM 2581.

With a working protocol established, we next sought to optimise electrocompetent cell preparation. To this end, we first tested washing the cells with glycerol (10%) instead of sucrose (300 mM), since glycerol is also widely used as a non‐ionic buffer in the preparation of electrocompetent cells in other Gram‐negative bacteria (Tu et al. 2016; Gonzales et al. 2013; Xu et al. 2022). A 3.8‐fold increase in electroporation efficiency was observed when electrocompetent cells were prepared in glycerol (10%) compared to sucrose (300 mM) (Figure 2b). Glycerol (10%) was thereafter adopted for subsequent experiments. In addition, we investigated how the growth stage of the culture at which cells are harvested for electrocompetent cell preparation affected electroporation efficiency. Surprisingly, cells harvested from 24 h cultures (late‐stationary phase) produced the highest transformation rates (4.5 × 10^3^ ± 1.5 × 10^3^ CFU/μg DNA), significantly outperforming cells harvested from 6 h (early‐logarithmic phase) and 14 h cultures (early‐stationary phase) (Figure 2c).

### 3.3 Influence of Salinity on Electroporation Efficiency

The susceptibility of halophilic bacteria to different antimicrobials has been shown to vary significantly with the salinity of the growth medium (Coronado et al. 1995). This phenomenon is particularly pronounced with aminoglycosides, including gentamicin, kanamycin and streptomycin, which target the bacterial ribosome and thus need to cross the lipopolysaccharide‐rich outer membrane, periplasmic space, and plasma membrane to access the ribosomes in the bacterial cytoplasm. For instance, in *H. elongata* , the minimum inhibitory concentration of kanamycin decreases drastically from 500 μg/mL at 7.5% NaCl to 125 μg/mL at 5% to 15.6 μg/mL at 2% (Coronado et al. 1995). These effects are thought to be caused by structural changes in the composition of the cell wall, thus facilitating the permeation of antibiotics into the cells.

**FIGURE 3:**
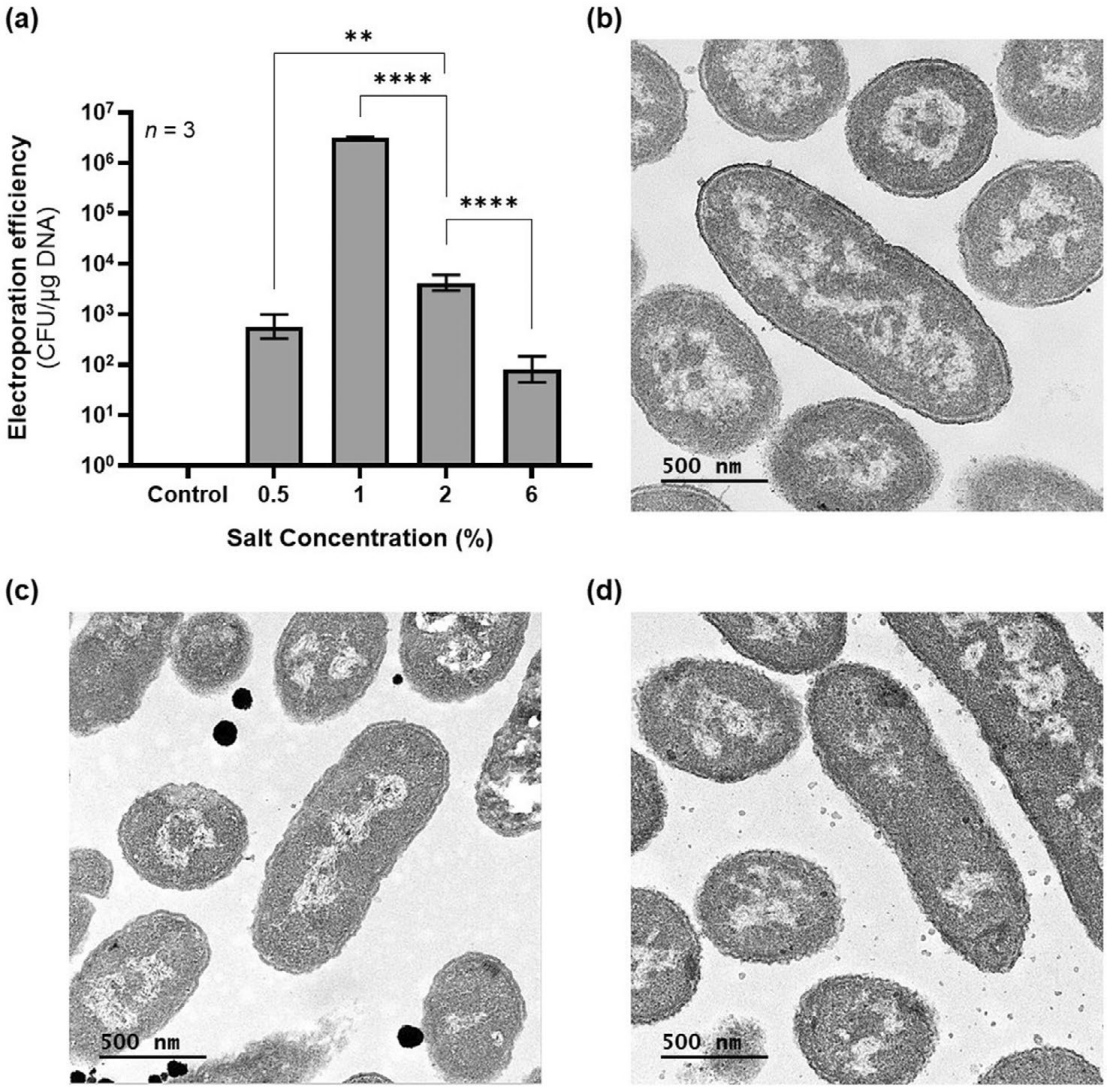
Effect of salinity on electroporation efficiency and cell morphology of *Halomonas elongata* DSM 2581. (a) Electroporation efficiencies from electrocompetent cells prepared from cultures grown in LB medium containing different concentrations of NaCl: 0.5%, 1%, 2% and 6%. Data shown represent geometric mean */ geometric SD factor from three biological replicates. Negative control experiments were performed by electroporating electrocompetent cells without the addition of plasmid pSEVA241. (**p < 0.01; ****p < 0.0001). (b) Transmission electron microscopy imaging of cells cultured for 24 h in LB medium containing 1% NaCl. (c) Transmission electron microscopy imaging of cells cultured for 24 h in LB medium containing 6% NaCl. (d) Transmission electron microscopy imaging of cells cultured for 14 h in LB medium containing 6% NaCl.

We therefore hypothesised that these structural changes could also affect plasmid uptake during electroporation. To test this theory, we compared the electroporation efficiency of electrocompetent *H. elongata* cells grown at 2% and 6% NaCl (Figure 3a). A 49‐fold increase in electroporation efficiency was observed at 2% NaCl compared to 6%, supporting the hypothesis that lower salinity enhances the cell's amenability to transformation. Encouraged by these results, we tested whether further decreasing salt concentration to 1% and 0.5% NaCl would further improve electroporation efficiency. At 1% NaCl, a 725‐fold improvement over the efficiency at 2% NaCl was observed (Figure 3a), with an average efficiency of 3.2 × 10^6^ ± 9.1 × 10^4^ CFU/μg DNA. However, efficiency decreased at 0.5% NaCl, likely due to reduced cell viability resulting from increased osmotic stress.

To investigate whether these results were linked to structural differences in the bacterial cell wall thickness, we performed transmission electron microscopy (TEM) on *H. elongata* grown at 1% and 6% NaCl (Figure 3b,c). While cell wall thickness did not seem to significantly differ between growth conditions (1%: 30.79 nm (*n* = 106); 6%: 31.02 nm (*n* = 108); *p* = 0.67) (Figure S3), cells cultivated at 1% NaCl exhibited a larger diameter compared to cells grown at 6% NaCl (1%: 640 nm (*n* = 154); 6%: 514 nm (*n* = 128); *p* < 0.0001). The increased surface area in low‐salt‐grown *H. elongata* cells may facilitate cell wall disruption during electroporation, thereby favouring plasmid uptake. In addition, TEM was also performed to investigate if the differences observed in electroporation efficiencies when cells were harvested at different growth stages could also be attributed to differences in cell size (Figure 3c,d). However, for cultures grown in 6% NaCl, cells harvested at 14 h were significantly larger than those harvested at 24 h (6%, 14 h: 587 nm (*n* = 145); 6%, 24 h: 514 nm (*n* = 128); *p* < 0.0001), thus suggesting that there are additional factors influencing transformation amenability.

### 3.4 Further Parameter Optimisation

**FIGURE 4:**
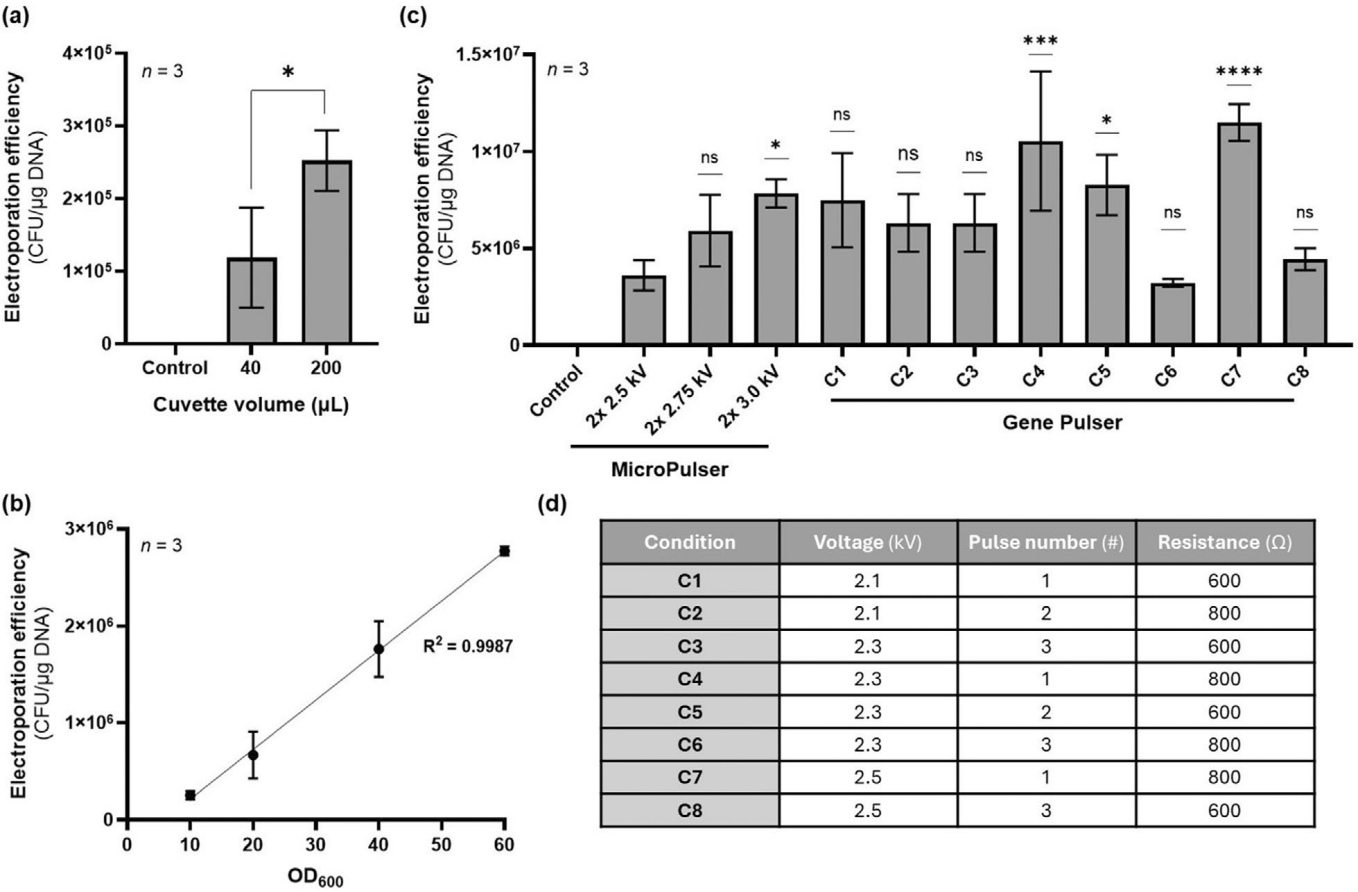
Further optimisation of electroporation parameters for *Halomonas elongata* DSM 2581. (a) Comparison of electroporation efficiencies using two different volumes of electrocompetent cell suspension: 40 μL vs. 200 μL. (b) Effect of cell concentration on electroporation efficiency. Electrocompetent cells were resuspended in glycerol (10%) at different optical densities: OD_600_ = 10, 20, 40, 60. (c) Comparison of electroporation efficiencies using two different electroporator systems—Bio‐Rad MicroPulser vs. Bio‐Rad Gene Pulser—under different electroporation conditions: Voltage, pulse number and resistance. (d) Table summarising the eight electroporation conditions tested in panel (c), resulting from the constrained mixed‐level orthogonal array design. Data shown represent mean ± standard deviation from three biological replicates. Negative control experiments were performed by electroporating electrocompetent cells without the addition of plasmid pSEVA241. (ns, not significant; *p < 0.05; ***p < 0.001; ****p < 0.0001).

To further enhance electroporation efficiency, the influence of other parameters was also investigated, including the concentration to which cells were resuspended in glycerol (10%) and the volume of cell suspension added to the electroporation cuvette (Figure 4). Reducing the electroporation cuvette volume from 200 to 40 μL led to a 47% decrease in transformation efficiency (Figure 4a). Likewise, resuspending the cells at lower concentration significantly decreased efficiency, as the highest electroporation efficiencies were achieved when cells were resuspended at OD_600_ = 60 prior to electroporation (Figure 4b). The fact that efficiency linearly decreased with cell concentration (*R* ^2^ = 0.9987) suggests that the number of viable cells added to the cuvette is a limiting factor of the method.

Since earlier experiments suggested that efficiency increased with increasing voltage (Figure 2a), we tested whether further increasing the voltage would continue this trend. Indeed, applying two pulses at 3.0 kV resulted in a 2.2‐fold improvement over the same pulse number at 2.5 kV (Figure 4c). Moreover, we wanted to investigate how further manipulating the other parameters involved in the electroporation process would affect efficiency. Up to this point, electroporation experiments were performed using a Bio‐Rad MicroPulser, which is limited to a fixed capacitance of 10 μF, and a 600 Ω resistor in parallel. Both capacitance and resistance influence the time constant (τ = RC) of the electroporation pulse, which represents the time over which voltage decay occurs (Castellví et al. 2017; Lurquin 2013). Thus, both parameters also influence the efficiency of the electroporation process. To enable control of these parameters, we employed a Bio‐Rad Gene Pulser, which allows independent manipulation of voltage, resistance and capacitance. To explore the combined effects of varying these parameters, a constrained mixed‐level orthogonal array experiment was designed, in which voltage (kV), pulse number (#) and resistance (Ω) were varied, while capacitance was kept at 25 μF. The eight different conditions tested are summarised in Figure 4d. However, when testing electroporation under these conditions, the formation of dense cell aggregates was observed during the recovery period, potentially due to cell lysis induced by the high‐energy pulses. We hypothesised that these effects were exacerbated by the structurally‐compromised cell wall of the cells grown under low‐salinity conditions. To mitigate this issue, the experiment was repeated using electrocompetent cells generated at 4°C, rather than at room temperature. This modification proved effective, as no dense cell aggregates were formed. Amongst the eight conditions tested, condition C7 (2.5 kV; 1 pulse, 800 Ω, 25 μF) produced the highest transformation efficiency, reaching 1.15 × 10^7^ ± 0.09 × 10^7^ CFU/μg DNA (Figure 4c).

### 3.5 Overcoming Restriction–Modification Systems to Improve Electroporation Efficiency

**FIGURE 5:**
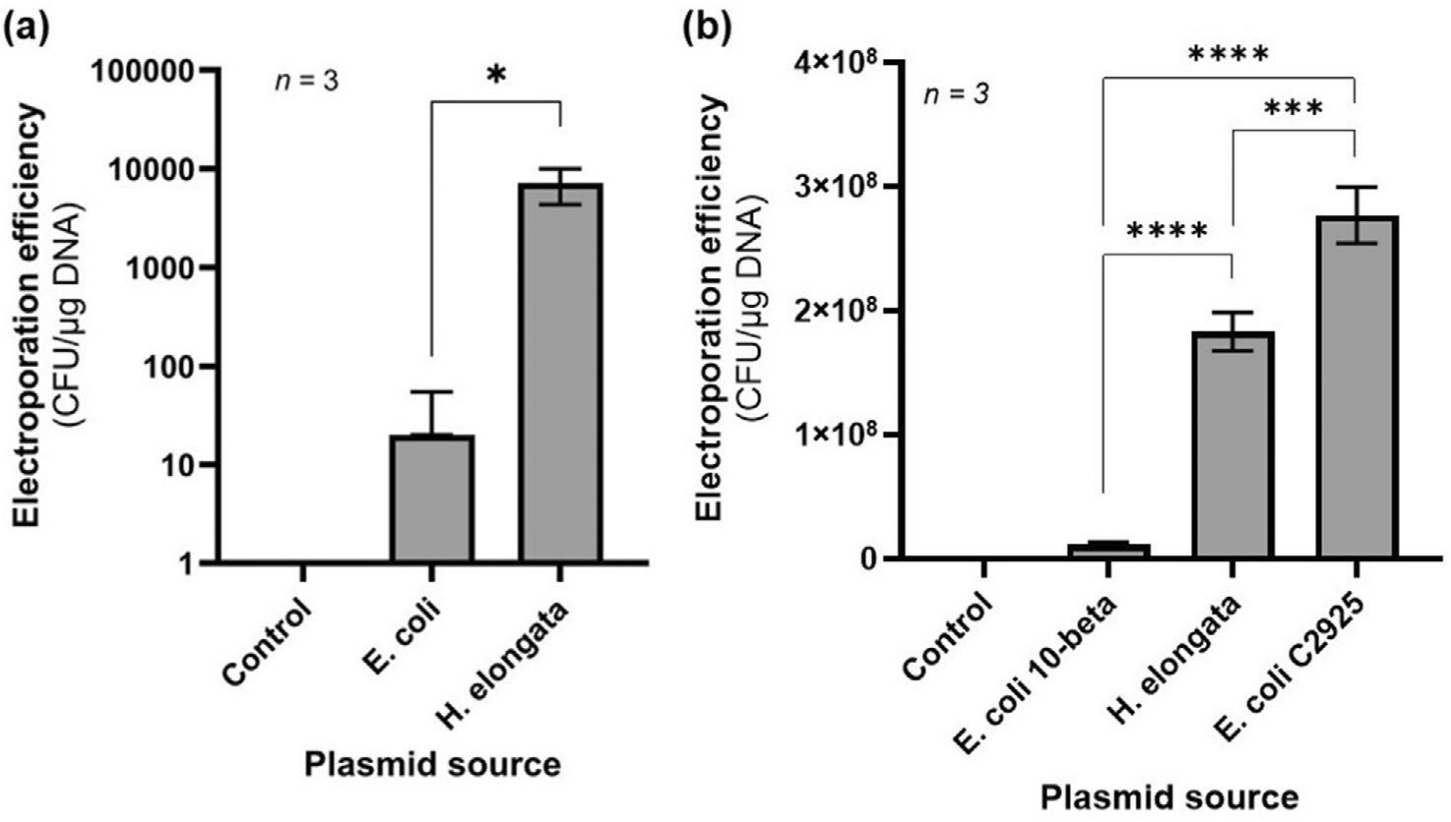
Effect of plasmid source on the electroporation efficiency of *Halomonas elongata* DSM 2581. (a) Electroporation efficiencies of pSEVA241 purified from either *E. coli* 10‐beta (NEB) or *H. elongata* DSM 2581. (b) Electroporation efficiencies of pSEVA231 purified from either *E. coli* 10‐beta (NEB), *E. coli* C2925 (NEB) or *H. elongata* DSM 2581. Data shown represent mean ± standard deviation from three biological replicates. Negative control experiments were performed by electroporating electrocompetent cells without the addition of plasmid pSEVA241 (a) or pSEVA231 (b) (*p < 0.05; ***p < 0.001; ****p < 0.0001).

Endogenous restriction‐modification (R–M) systems are known to limit transformation efficiency by degrading foreign DNA that carries non‐native methylation patterns (Ren et al. 2022; Vajente et al. 2024). In our experiments, plasmids were initially purified from *E. coli* 10‐beta (NEB), which expresses the *dam* and *dcm* methyltransferases. The methylation patterns produced by these enzymes can be recognised by R–M systems of *H. elongata* DSM 2581, potentially leading to plasmid degradation and consequently reduced transformation efficiency. To assess whether this was the case, we electroporated pSEVA241 plasmids purified from *H. elongata* DSM 2581 and compared electroporation efficiency to that of plasmids purified from *E. coli* 10‐beta. Since plasmids purified from *H. elongata* should display the bacterium's own native methylation profile, they are expected to evade the restriction systems. Indeed, a 359‐fold increase in electroporation efficiency was observed when pSEVA241 was purified from *H. elongata* (Figure 5a), suggesting that the *dam*/*dcm* methylation from *E. coli* 10‐beta triggers the host's restriction machinery.

To overcome this issue, previous studies have resorted to expressing the native methyltransferases of the host organism into methyltransferase‐deficient *E. coli* strains, enabling plasmid propagation without triggering the host R–M systems (Ren et al. 2022; Zhang et al. 2012). Following this approach, we tested electroporating *H. elongata* with plasmids purified from the *dam* ^*−*^/*dcm* ^*−*^ *E. coli* C2925 (NEB). However, this strain does not have *recA* knocked out, and the pSEVA241 plasmid harbours a recombination‐prone pRO1600/ColE1 origin of replication (Kolodner et al. 1985; Mcgraw and Marinus 1980; Marinus and Morris 1974), thus making it susceptible to plasmid concatemerisation and instability (Maucksch et al. 2013; Subia and Kogoma 1986; Viret et al. 1991), which could significantly affect the reliability of any electroporation efficiency tests. To eliminate this confounding variable, subsequent electroporation experiments were performed using plasmid pSEVA231 instead, which carries the broad‐host range pBBR1 origin of replication, which is not recombination‐prone (Antoine and Locht 1992).

Before introducing *H. elongata* DSM 2581 methyltransferases in *E. coli* C2925, we first compared electroporation efficiencies of pSEVA231 plasmids purified from either *E. coli* 10‐beta, *E. coli* C2925 or *H. elongata* DSM 2581. Surprisingly, the highest electroporation efficiency was achieved using pSEVA231 purified from *E. coli* C2925. Under this condition, electroporation yields reached 2.8 × 10^8^ ± 0.2 × 10^8^ CFU/μg DNA, the highest transformation efficiency ever reported for any *Halomonas* species to date (Figure 5b). This efficiency level represents a 24‐fold increase over that observed for pSEVA231 purified from *E. coli 10‐beta*, suggesting that the absence of DNA methylation in the plasmid was sufficient to bypass the defence systems responsible for recognising and degrading foreign DNA in *H. elongata* . Importantly, this suggests that generating an *E. coli* strain that emulates the same methylation pattern as *H. elongata* should not be necessary, thus significantly simplifying plasmid preparation for transformation. Interestingly, while pSEVA231 purified from *H. elongata* itself also produced higher electroporation efficiencies than the methylated pSEVA231 purified from *E. coli* 10‐beta, it yielded a 1.5‐fold lower efficiency compared to that of pSEVA231 from *E. coli* C2925 (Figure 5b). However, this discrepancy is more likely attributable to systematic variations that arise during the electroporation process, as well as differences in the concentration and purity of the plasmids obtained from different strains, rather than to biological differences.

### 3.6 Electroporation of Other *Halomonas* Species

Having established an efficient electroporation protocol for *H. elongata* DSM 2581, we next investigated whether this method could be adapted for other industrially relevant *Halomonas* species. To this end, *Halomonas boliviensis* LC1 and *Halomonas campaniensis* LS21 were selected due to their potential as industrial biotechnology chassis (Coimbra et al. 2025).

**FIGURE 6:**
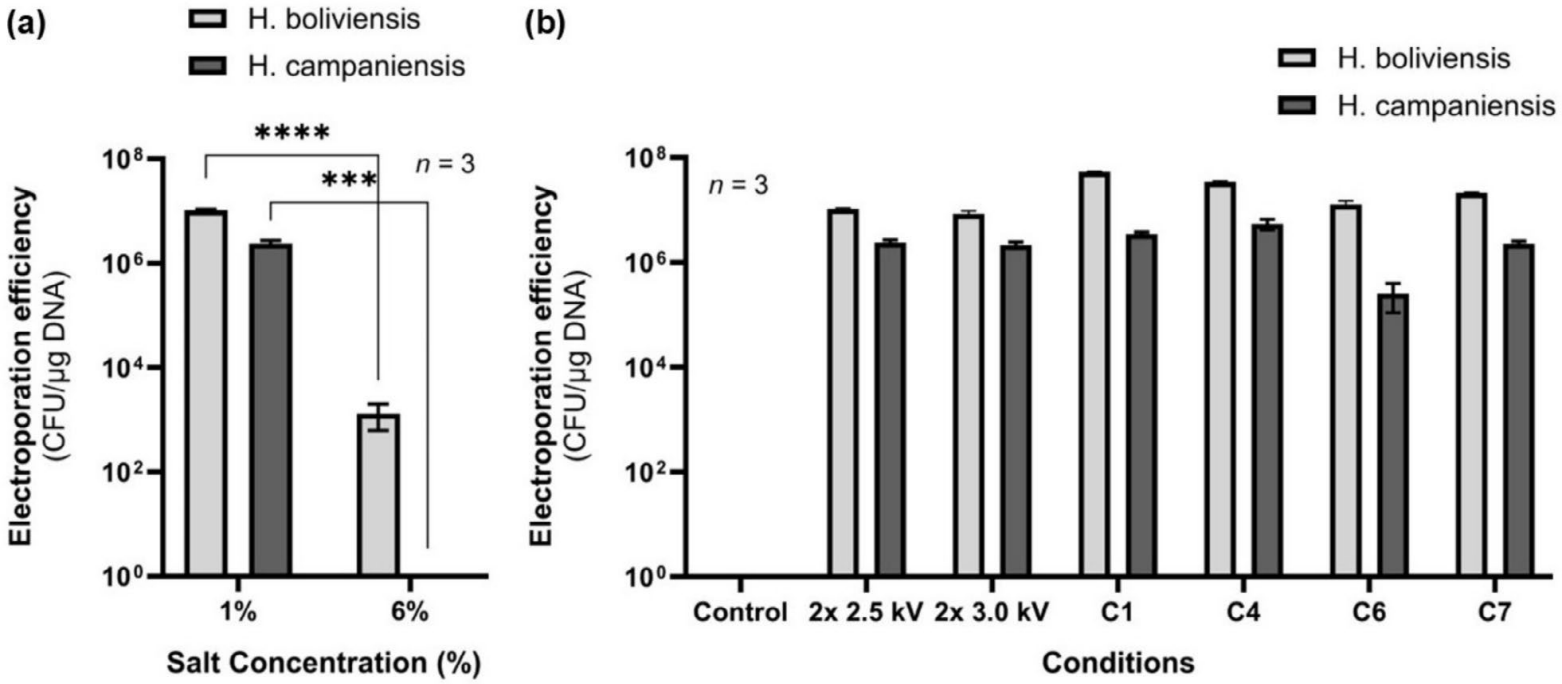
Electroporation of *Halomonas boliviensis* LC1 and *Halomonas campaniensis* LS21. Electroporation efficiencies of *H. boliviensis* LC1 and *H. campaniensis* LS21 transformed with pSEVA231 purified from either *E. coli* C2925 (NEB) (a) Comparison of electroporation efficiencies from electrocompetent cells prepared from cultures grown in LB medium containing different concentrations of NaCl: 6% vs. 1%. (b) Comparison of electroporation efficiencies using two different electroporator systems—Bio‐Rad MicroPulser vs. Bio‐Rad Gene Pulser—under varying electroporation conditions: Voltage, pulse number and resistance. Parameters for conditions C1, C4, C6 and C7 are detailed in Figure 4d. Data shown represent mean ± standard deviation from three biological replicates. Negative control experiments were performed by electroporating electrocompetent cells without the addition of plasmid pSEVA231. (***p < 0.001; ****p < 0.0001)

Given the strong effect of salinity on *H. elongata* electroporation efficiency, we first tested whether this effect was also observed in *H. boliviensis* and *H. campaniensis* . When grown in high‐salt (6% NaCl) medium, both strains exhibited a drastic reduction in efficiency compared to low‐salt (1% NaCl) medium when electroporated with pSEVA231 purified from *E. coli* C2925 (Figure 6a). In fact, no colonies were obtained for *H. campaniensis* LS21 when cells were cultured at 6% NaCl. Electroporation efficiencies for both strains were then optimised by testing a range of voltages, pulse numbers and resistance settings, as previously performed for *H. elongata* . The highest electroporation efficiency for *H. boliviensis* LC1 was 5.3 × 10^7^ ± 0.1 × 10^7^ CFU/μg DNA, obtained under condition C1 (2.1 kV; 1 pulse; 600 Ω; 25 μF), while for *H. campaniensis* LS21 a maximum efficiency of 5.4 × 10^6^ ± 1.2 × 10^6^ CFU/μg DNA was achieved under condition C4 (2.3 kV; 1 pulse; 800 Ω; 25 μF) (Figure 6b). These results demonstrate that the optimised protocol for *H. elongata* can also be effectively applied to efficiently electroporate other *Halomonas* species.

## 4 Discussion

In this study, we developed a highly efficient electroporation protocol for the hard‐to‐transform *Halomonas elongata* DSM 2581. By optimising competent cell generation and electroporation parameters, and using plasmid DNA purified from methylation‐deficient *E. coli* , a maximum efficiency of 2.8 × 10^8^ ± 0.2 × 10^8^ CFU/μg DNA was obtained. This represents the highest electroporation efficiency ever reported for any *Halomonas* species to date (Harris et al. 2016; Jung et al. 2024; Xu et al. 2022; Coimbra et al. 2025). In fact, in 2024, Jung et al. reported that they were unable to electroporate this strain. Furthermore, the efficiencies that were obtained with the optimised protocol are comparable to those of other well‐established model microorganisms, such as *E. coli* and *P. aeruginosa* (Calvin and Hanawalt 1988; Choi et al. 2006).

One of the key findings of this study followed the investigation of the growth stage at which cells were harvested. While typically electrocompetent bacterial cells are prepared from cultures grown to early‐ to mid‐logarithmic phase (O'Callaghan and Charbit 1990; Cui et al. 2015; Alegre et al. 2004; Lessard 2013), we observed that extending incubation until the late‐stationary phase (24 h) drastically increased electroporation efficiency. One potential drawback of this approach, however, is the increased risk of accumulation of spontaneous mutations resulting from the extended incubation period, which would be undesirable for further downstream applications. Nevertheless, further research on this matter would be required to evaluate the extent of this risk.

Across all three *Halomonas* strains studied, the salt concentration at which cells were initially cultured strongly influenced electroporation efficiency. TEM imaging of *H. elongata* grown at 1% and 6% NaCl suggested that the larger cell size observed at low salinity may be correlated to improved transformation efficiency. This observation is consistent with the work of Miguelez and Gilmour (1994), who previously reported a 1.3‐fold increase in intracellular volume of *H. elongata* DSM 2581 grown at 1% NaCl compared to 8% NaCl. However, while harvesting cells at 24 h enhanced electroporation efficiency compared to 14 h, a decrease in cell size was observed at the later time point, suggesting that other additional physiological or structural factors influence the cell's ability to uptake plasmids. Indeed, whole‐cell phospholipid analysis has revealed significant changes in the phospholipid‐to‐protein ratio and phospholipid composition between *H. elongata* cultures grown at 0.3% and 8% NaCl (Vreeland et al. 1984). Importantly, because electroporation efficiency proved highest at 1% NaCl, future genetic manipulation work should preferentially be carried out under low‐salt conditions, in both *H. elongata* as well as other *Halomonas* species. However, this strategy may limit the electroporation efficiency levels reached in strains unable to tolerate very low salt concentrations. A potential solution to address this issue would be to perform adaptive evolution experiments, whereby such strains would be gradually cultured in lower salt concentrations.

Restriction–modification (R–M) systems can play a major role in decreasing transformation efficiency in bacteria. Indeed, we observed a 359‐fold and 16‐fold increase in electroporation efficiency of pSEVA241 and pSEVA231, respectively, when these plasmids were purified from *H. elongata* DSM 2581 rather than *E. coli* 10‐beta. The greater fold improvement observed for pSEVA241 could reflect a greater susceptibility of this plasmid to the host defence systems, as pSEVA241 contains a higher number of potential Dam (17) and Dcm (12) methylation sites relative to the 9 present in pSEVA231. At the same time, it is also possible that the use of the optimised protocol for the electroporation of pSEVA231 could have decreased the negative influence of the host's defence systems on the efficiency levels. Notably, efficiency levels were restored by transforming *H. elongata* with pSEVA231 purified from *dam* ^*−*^ */dcm* ^*−*^ *E. coli* C2925, suggesting that non‐methylated DNA is capable of evading the host's defences. This phenomenon has also been previously reported in other halophilic organisms. For instance, Holmes et al. (1991) observed a 10^3^‐fold improvement in transformation efficiency of the archaeon *Haloferax volcanii* when plasmid DNA was purified from *dam*/*dcm* methylation‐deficient *E. coli* JM110.

The use of *E. coli* lacking *dam*/*dcm* methylation for plasmid preparation provides a simple yet effective strategy to overcome the host defence systems, thus facilitating and streamlining future genetic manipulation work in *Halomonas elongata* . This contrasts with other more laborious approaches often required to evade the native R–M systems in other non‐conventional microorganisms, such as targeted deletion of R–M systems (Grybchuk‐Ieremenko et al. 2025), heterologous expression of host‐derived methyltransferases in methyltransferase‐deficient *E. coli* (Monk et al. 2015; Zhang et al. 2012; Riley et al. 2019), or mimicking host DNA methylation patterns on plasmid DNA through an in vitro transcription‐translation (IMPRINT) of host methyltransferases (Vento et al. 2024). However, it should be noted that because R–Ms and other bacterial defence systems will vary across different *Halomonas* species, the effectiveness of using unmethylated plasmid DNA to improve electroporation efficiency might vary substantially between species, although the extent of that influence would need to be studied on a strain‐by‐strain basis.

The most plausible explanation for the increased electroporation efficiency observed in *H. elongata* DSM 2581 when using unmethylated plasmid DNA is the presence of a Type IV R–M system. Type IV nucleases preferentially cleave methylated or otherwise chemically modified DNA and typically exhibit low sequence selectivity (Loenen and Raleigh 2014). Although no Type IV system is currently annotated for *H. elongata* in the REBASE database (Roberts et al. 2023), such systems are often difficult to detect due to their extensive sequence diversity and structural heterogeneity (Oliveira et al. 2014; Readshaw et al. 2025; Anton and Roberts 2023). Thus, the presence of an uncharacterised Type IV system in *H. elongata* cannot be excluded. In addition to classical R–M systems, other more recently described methylation‐associated bacterial defence mechanisms, such as BREX (Goldfarb et al. 2015) and DISARM (Ofir et al. 2018), may also influence transformation efficiencies. However, no defence systems present in *H. elongata* DSM 2581 identified by the PADLOC (Payne et al. 2022) or DefenseFinder (Tesson et al. 2022, 2024) databases could be directly linked to our observations. Therefore, future work should focus on methylome profiling of *H. elongata* DSM 2581, alongside systematic identification and functional characterisation of its foreign DNA‐targeting defence systems, particularly those involved in the recognition and degradation of methylated plasmid DNA.

Excitingly, the protocol developed for *H. elongata* proved readily transferable to two other industrially relevant *Halomonas* strains. An efficiency of 5.4 × 10^6^ ± 1.2 × 10^6^ CFU/μg DNA was obtained for *H. campaniensis* LS21, a particularly promising strain, having been engineered to grow to 34 g/L dry cell weight (DCW), while accumulating 60% PHB in an open fermentation in a 7.5‐L bioreactor (Jiang et al. 2017). Moreover, despite *H. boliviensis* LC1 having been observed to naturally produce high levels of both PHB and ectoine (Quillaguamán et al. 2008; Van‐Thuoc et al. 2010), as well as having a broad‐substrate metabolism (Coimbra et al. 2025), no genetic manipulation studies have been performed on it so far. As such, the 5.3 × 10^7^ ± 0.1 × 10^7^ CFU/μg DNA efficiency levels reached using our protocol should encourage such studies in the future. Likewise, the ease of adapting the electroporation protocol from *H. elongata* to the other two *Halomonas* strains without need for major optimisation work should encourage further work in other underexplored strains as well.

Electroporation offers major advantages over existing conjugation methods, which are restricted to conjugative/mobilisable plasmids, impose DNA size limitations, and are incompatible with the genetic manipulation work using linear DNA or ribonucleoproteins. In contrast, electroporation is more versatile, has higher throughput, and is thus better suited for experiments requiring large library screening. Hence, our work in establishing an efficient and transferable electroporation protocol should facilitate genetic manipulation work and unlock the biotechnological potential of *Halomonas* species.

## Author Contributions

**André A. B. Coimbra:** conceptualization, methodology, data curation, investigation, writing – original draft, validation, writing – review and editing. **Ian J. White:** methodology, data curation, investigation, writing – review and editing. **Leonardo Rios‐Solis:** conceptualization, project administration, resources, supervision, writing – review and editing.

## Funding

This work was supported by Engineering and Physical Sciences Research Council (EP/T517793/1, EP/W524335/1).

## Ethics Statement

The authors have nothing to report.

## Conflicts of Interest

The authors declare no conflicts of interest.

## Supporting information


**Figure S1:** mbt270285‐sup‐0001‐FigureS1.jpg.

**Figure S2:** mbt270285‐sup‐0002‐FigureS2.jpg.

**Figure S3:** mbt270285‐sup‐0003‐FigureS3.jpg.

## Data Availability

The data that support the findings of this study are available from the corresponding author upon reasonable request.
