# Supplementary figures and images for: Developing High‐Efficiency Electroporation Protocols for Hard‐To‐Transform Halomonas spp
Source: Microb Biotechnol. 2025 Dec 21;18(12):e70285. doi: 10.1111/1751-7915.70285 (PMC12719606; doi:10.1111/1751-7915.70285)

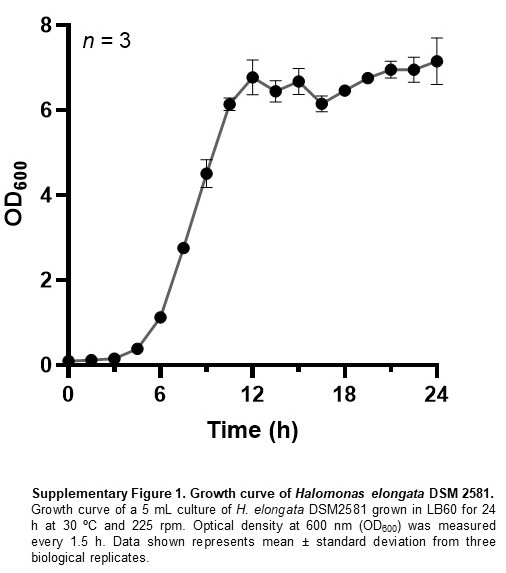

Supplement: Supplementary file 1 — Figure S1: mbt270285‐sup‐0001‐FigureS1.jpg. [file MBT2-18-e70285-s001.jpg]

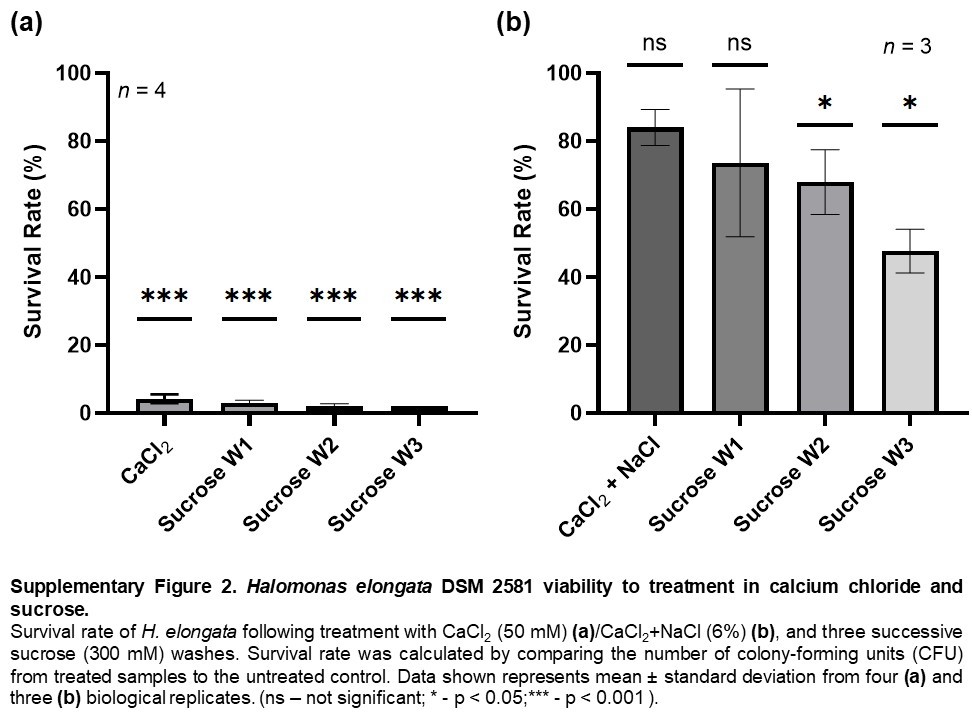

Supplement: Supplementary file 2 — Figure S2: mbt270285‐sup‐0002‐FigureS2.jpg. [file MBT2-18-e70285-s002.jpg]

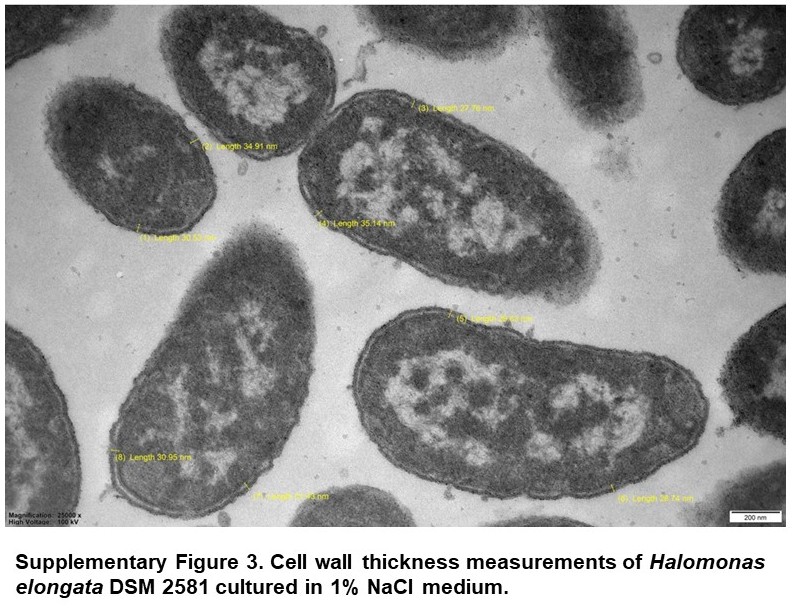

Supplement: Supplementary file 3 — Figure S3: mbt270285‐sup‐0003‐FigureS3.jpg. [file MBT2-18-e70285-s003.jpg]
